# Device for lengthening of a musculotendinous unit by direct continuous traction in the sheep

**DOI:** 10.1186/1746-6148-8-50

**Published:** 2012-05-02

**Authors:** Matthias A Zumstein, Eric Frey, Brigitte von Rechenberg, Robert Frigg, Christian Gerber, Dominik C Meyer

**Affiliations:** 1 Dept. of Orthopedics, University of Zurich, Balgrist, Zürich, 8008, Switzerland; 2 Chief Technology Officer, Synthes GmbH, Muracherstrasse 3, 2544, Bettlach, Switzerland; 3 Dept of Orthopedics, University of Berne, Inselspital, Bern, 3010, Switzerland; 4 Musculoskeletal Research Unit (MSRU), Equine Hospital, Vetsuisse Faculty, University of Zurich, Winterthurerstrasse 260, 8057, Zurich, Switzerland; 5 Competence Center for Applied Biotechnology and Molecular Medicine (CABMM), Vetsuisse Faculty, University of Zurich, Winterthurerstrasse 190, 8057, Zürich, Switzerland

**Keywords:** Muscle, Sheep, Retraction, Fatty infiltration, Atrophy, Pennation angle, Continuous traction

## Abstract

**Background:**

Retraction, atrophy and fatty infiltration are signs subsequent to chronic rotator cuff tendon tears. They are associated with an increased pennation angle and a shortening of the muscle fibers in series. These deleterious changes of the muscular architecture are not reversible with current repair techniques and are the main factors for failed rotator cuff tendon repair. Whereas fast stretching of the retracted musculotendinous unit results in proliferation of non-contractile fibrous tissue, slow stretching may lead to muscle regeneration in terms of sarcomerogenesis. To slowly stretch the retracted musculotendinous unit in a sheep model, two here described tensioning devices have been developed and mounted on the scapular spine of the sheep using an expandable threaded rod, which has been interposed between the retracted tendon end and the original insertion site at the humeral head. Traction is transmitted in line with the musculotendinous unit by sutures knotted on the expandable threaded rod. The threaded rod of the tensioner is driven within the body through a rotating axis, which enters the body on the opposite side. The tendon end, which was previously released (16 weeks prior) from its insertion site with a bone chip, was elongated with a velocity of 1 mm/day.

**Results:**

After several steps of technical improvements, the tensioner proved to be capable of actively stretching the retracted and degenerated muscle back to the original length and to withstand the external forces acting on it.

**Conclusion:**

This technical report describes the experimental technique for continuous elongation of the musculotendinous unit and reversion of the length of chronically shortened muscle.

## Background

Chronic rotator cuff tears lead to retraction of the musculotendinous unit, atrophy and hitherto irreversible fatty infiltration of the rotator cuff muscles [1-6]. In the sheep, muscle retraction and fatty infiltration were shown to be related to a pronounced change in the pennation angle of the infraspinatus muscle with consequent shortening but absence of degeneration of the muscle fibres [7-9]. This shortening of the fibres may reduce muscle mass by >50% as a result of a reduction of sarcomeres in series [8]. Physiologically, these muscles loose their contractile strength, range of contraction and elasticity [7,8,10-12]. These changes are not reversible with current tendon repair techniques.

Muscle homeostasis is regulated by different mechanical signals [13,14], primarily by active or passive tension applied on the tissue, respectively. In response to an increase of length, the functional demand of the tissue may be higher and the muscle tissue may increase in size by appearance of embryonic tissue, indicating a proliferative response [15-17]. Skeletal muscle reacts differently following repeated tensile strains. Fast stretching has been reported to potentially disrupt muscle fibers and to lead to a proliferation of non-contractile tissue [18]. Conversely, slow, continuous stretching results in skeletal muscle hypertrophy by an increase in absolute muscle mass due to a moderate increase of myofiber cross sectional area [18,19] and by a substantial longitudinal increase of sarcomere in series [19-21]. It is not yet known, however, whether these mechanisms are effective in muscle, which is structurally altered due to chronic tendon tearing.

To test our hypothesis that continuous traction might allow restoration of structure and function of chronically retracted muscle, an in-vivo technique had to be developed to apply continuous traction to a retracted musculotendinous unit. It is the purpose of this paper to present a new experimental tool and corresponding technique, which allows applying tension to a chronically retracted musculotendinous unit in the sheep model.

## Methods

**Figure 1:**
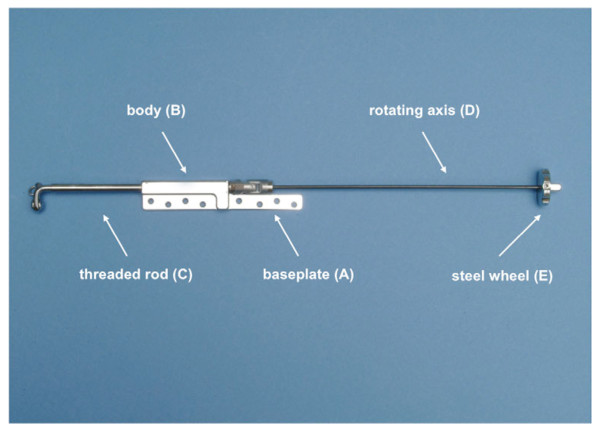
**Tensioning device with holes for locking screws through the baseplate(A).** Through axial rotation on the steel wheel (E) the threaded rod (C) exits the opposite side of the body (B) and thereby elongates the muscle tendon unit, which is fixed to the end of the threaded road by sutures.

An apparatus called “tensioner” allowing application of tension to the infraspinatus muscle-tendon-unit without necessitating limb immobilization was designed:

Tensioner (Figure 1):

The tensioning device (“tensioner”) comprised a base plate (A) for fixation, which was welded to a longitudinal body (B). The body of the tensioner housed a threaded rod (C, threads hidden in the body), which was driven out of the body (B) by turning a rotating axis (D) which entered the body (B) on the opposite side of the rod. The rotating axis was made of Nitinol® and had a length of 155 mm. At the end of the rotating axis, a steel wheel of 20 mm diameter was fixed to allow lengthening of the threaded rod. Turning of the rotating axis by 360° results in an extrusion of the rod by 1 mm. A clicking mechanism gives a small resistance after every full turn (1 mm, 360°) of the axis and inhibits unwanted passive re-rotation of the rod. The rod displaces rotating clockwise and that the click mechanism prevents counter clockwise rotations. This axis (D) is connected to the body axis (B) with a universal (or Cardan) joint to allow angulations of axis (D) relative to the body axis (B). The steel wheel (E) mounted on rotating axis D is external to the sheep’s body. The Cardan joint is protected from spontaneous scarring and tangling with adjacent soft-tissue with a silicon tube. The apparatus is fixed on the scapular spine of the sheep with locking screws, which prevent angular shifting of the baseplate.

Two different tensioners were manufactured and implanted during experiments. Tensioner No.1 was developed in collaboration with the AO, Development Center (ADI, Davos, Switzerland). For fixation of the tensioner in-vivo experiments, device No. 1 consisted of a base plate welded to the body of the tensioner. The frame was then fixed to the scapular spine using a maximum of four screws in the fixation plate (Figure 1).

**Figure 2:**
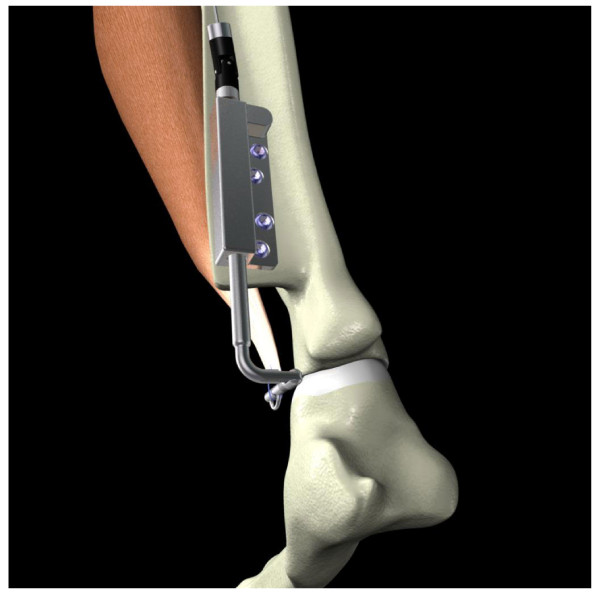
Tensioner No.1 mounted on the scapular spine of the sheeps’ shoulder with 4 holes for fixed angle locking screws and a bent threaded rod ‘end’.

**Figure 3:**
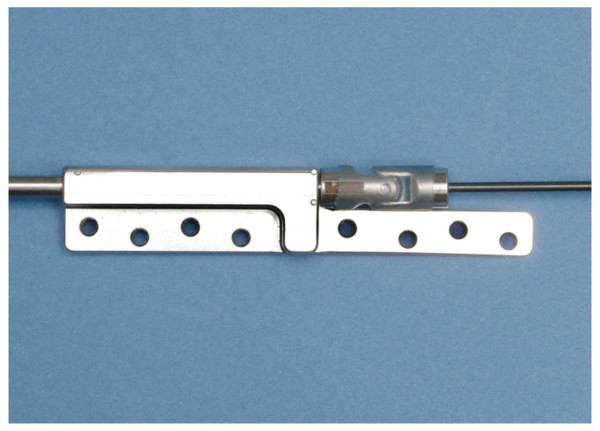
Tensioner No. 2 with expansion of the baseplate and 8 possible screw placements.

The threaded rod allowed expanding the device by 35 mm. The distal part of the threaded rod was bent twice to bring its end in line with the muscle tendon. The end of the 3 mm diameter rod was equipped with an eyelet (3.5 mm radius; 1 mm thick) to secure the sutures holding the tendon to it during the “in-vivo-experiments”. Because of cut outs of four baseplates out of the scapular spine with tensioner No. 1, it was changed to the technically improved tensioner No. 2 over the course of the experiment. This tensioner No. 2 was performed in collaboration with Synthes (Synthes GMBH, Bettlach, Switzerland). The second generation tensioner consisted of a longer base plate with eight screw holes for fixation of the base plate to the scapular spine (Figure 3).

The same diameter, thickness and materials were used for the base plate, body, threaded rod, pin and loop of the tensioner No. 2. (produced by Synthes GmbH, Solothurn, Switzerland). To reach more stability the multiaxial joint was made of stainless steel. Both, the Nitinol®-rod and the steel wheel had a higher diameter of 2 mm, and 32 mm respectively.

In vitro experiments:

### Expansion force generation

Axial forces developed actively with both tensioners by extruding the threaded rod from the body, were measured using an Instron Universal Testing Machine 4202 (Instron Limited, High Wycombe, HP12 3SY, England) with a 5kN load cell, mounted to the cross-bar. The sample was fixed to the lower table of the machine, using a vice holding the body of the scapula. The mean maximal axial force that can be developed by hand with each tensioner was tested by three different examiners (EF, DM, and MZ).

### Cyclic testing

**Figure 4:**
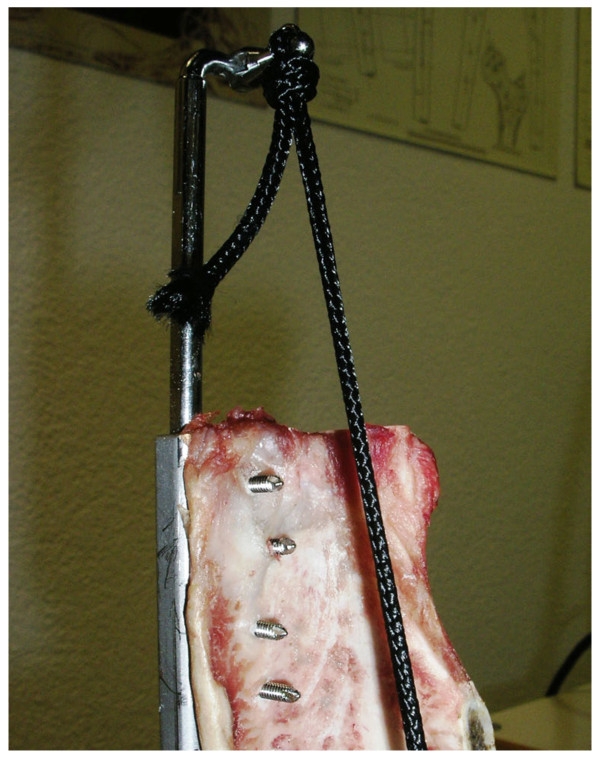
The setup of the loading direction made up in line with the sheeps’ infraspinatus muscle.

Three tensioner units of versions No. 1 and No. 2 were tested with cycling loading experiments. The tensioners were fixed on the scapular spine with 4 screws in tensioner No. 1 and six screws in tensioner No. 2, respectively. They were mounted on an Instron Universal Testing Machine 4202 (Instron Limited, High Wycombe, HP12 3SY, England) with the adjustable fixation device, which allowed adjustment of direction of pull. The direction of load application corresponded to the axis of the infraspinatus muscle in vivo (Figure 4).

Each tensioner was tested individually. To test the most vulnerable configuration, the tensioner was maximally extended to gain the longest lever arm. Cyclic loading was performed to simulate the in vivo conditions best, similar to prior studies [10,22]. In both tensioners, a constant base load of 20 N was applied. For both tensioners, one hundred cycles of 10 N (lower limit) to 100 N (upper limit) were applied with a crosshead speed of 20 mm per minute and showed a sinus load curve. Further continuous increase of the upper load limit was applied using the same setup and cycles until failure occurred. The ultimate tensile strength and the modes of failure were recorded for each tensioner.

In vivo experiments:

In-vivo experiments were performed with 12 white alpine sheep. The mean age of the sheep at the beginning of the experiments was 15.5 months, with a range from 14 to 17 months. The mean weight at the beginning of the experiment was 45.2 ± 4.0 kg. All experiments were conducted according to the Swiss law of animal welfare and use for animal experiments (permission number ZH nr. 193/2004).

### Implantation

The surgical approach was performed using a curved incision over the lateral aspect of the acromion [7] under general anaesthesia [23]. According to the previous study [7], the infraspinatus was released using an osteotomy of the greater tuberosity. Two Fibre wire No. 5 sutures (Arthrex, Naples, FL) were passed in a figure-of-eight configuration through tendon and bone. The sutures and the greater tuberosity were wrapped into a 5 cm long silicone rubber tube (Silicone Penrose drain tube, 12 mm, Fortune medical instrument corp. Taipei, Taiwan). The tube was closed with non-resorbable suture at its end to prevent spontaneous healing. After a retraction and degeneration time of 40 weeks, the tensioner was implanted using the same approach. The locking-fixation-plate was mounted on the scapular spine, at the side of the supraspinatus muscle, with 4 locking plate screws in tensioner No.1 and 7 locking plate screws in tensioner No. 2. Tensioner No. 1 was primary implanted in 10 and tensioner No. 2 in two sheep. To prevent infection, the rotational axis was passed through a 150 mm subcutaneous tunnel before exiting through the skin. The sutures were knotted with repeated surgical knots on the eyelet of the treaded expanding rod and a basic tension of 20 N was applied.

#### Stretching

By turning the steel wheel 360° once a day the tensioner pulled the tendon axially by a distance of 1 mm/day towards its original insertion.

#### Radiographic assessment

To document the elongation of the musculotendinous unit, computer tomograms were performed after implantation and every 14 days under general anesthesia. The position of the tensioner and the greater tuberosity were measured in relation to the midglenoidal plane.

#### Tensioner removal

Subsequent to the elongation protocol, the tensioner was removed and the greater tuberosity was reattached as near as possible to its original insertion using 3.5 mm cortical bone screws and the previously prepared sutures. According to a previous study [7], the fixation was augmented by two Fiber Wire No. 2 sutures (Arthrex, Naples, FL). Postoperatively, rehabilitation included prevention from lying down by using a loose suspension net [7,11,24]. To avoid full weight bearing, a ball was attached to the sheep’s’ claw for six weeks postoperatively.

## Statistics

Boxplots and unpaired t-tests were performed to evaluate possible differences between the applied forces, displacements and maximal failure load of the two tensioners with SPSS version 15.0 for Windows (SPSS, Chicago, Illinois). The level of significance was set at p < 0.05.

## Results

**Figure 5:**
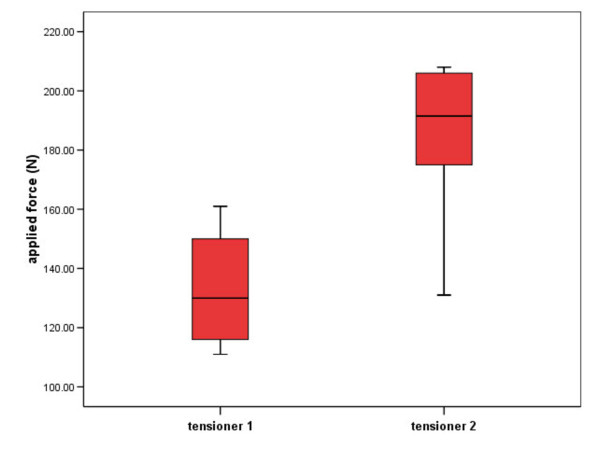
Mean maximal expansion forces of both No. of tensioners applied by the examiners in Newton (N).

Expansion force generation (Figure 5)

The maximal possible load applied by the three examiners was 133 ± 19.03 N with tensioner No.1 and 183.3 ± 28.71 N with tensioner No. 2. The difference between the maximal possible expansion forces of the two tensioners was significant (p = 0.017). Boxplots of expansion forces applied to both tensioners are displayed in Figure 5.

**Figure 6:**
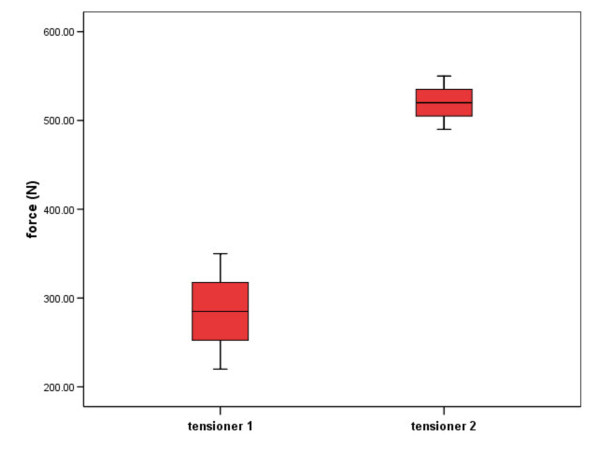
Mean maximal load to failure after 100 cycles of cyclic loading of bony fixation in both tensioners in Newton (N).

Stability in cyclic testing (Figure 6)

**Figure 7:**
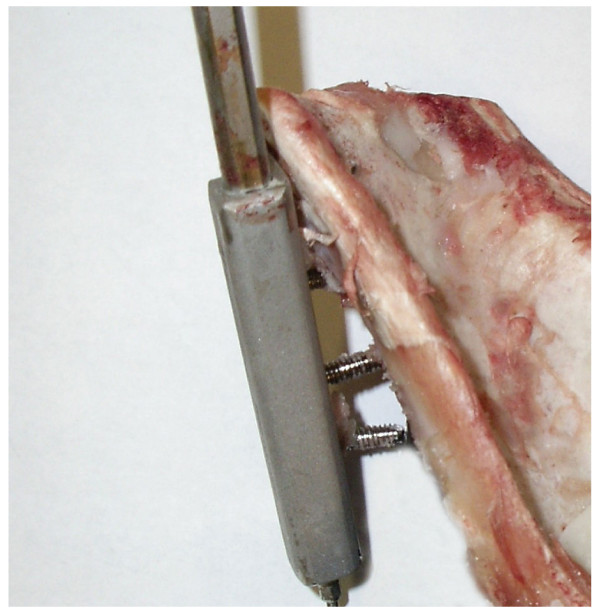
Pull-out of a tensioner No.1 through the scapular bone.

According to a previous study [24], the mean tension-range on the tendon of the infraspinatus sheep muscle ranges between 50 - 100 N during daily activities of 48 hours. The maximal tension registered on the tendon was 310 N. For both tensioner versions No.1 and No. 2, the base load was 10 N up to the upper limit of 100 N, and then increased up to the ultimate tensile strength up to 300 N respectively. Cyclic loading of 100 cycles showed slight bending of the threaded rod but no failure of the threaded rod in either tensioner type. Two of the three tensioners No. 1 failed by screw pull-out from the scapular bone (Figure 7), and one by fracture of the scapular spine. All tensioners No. 2 failed by fracture of the scapula. The maximal load to failure forces was significantly higher in tensioner No. 2 (285 ± 37.5 for tensioner 1, and 520 ± 30 for tensioner 2 respectively; p = 0.005).

### In vivo experiments

The specific results are thoroughly reported and published separately in a paper describing the specific biological findings [25]. Elongation of the musculotendinous unit to its original length was successful in eight of the twelve sheep and the tendon could in part or entirely be brought back to its original length. Only in two of the eight sheep, elongation was successful with tensioner No. 1. In four sheep, the tensioner No. 1 had to be exchanged to tensioner No. 2 because of screw pull-out from the bone (3 sheep) and one additional breakage of the Nitinol® rotating axis (1 sheep). Therefore, the design and its fixation on the scapular spine have been modified during the investigation. The number of fixation points has been changed from initially four to maximal eight screw-holes and the diameter of the rotating axis behind the tensioners’ body has been increased from 1 to 2 mm. In two of the eight successful sheep elongation was successful subsequent to primary implantation of tensioner No. 2. No failure was observed using the six tensioners No. 2.

However, in the remaining four sheep the elongation failed with tensioner No. 1: Of these, three sheep had an infection developed with abscess formation around the tensioner and the tendon-bone interface and the tendon disintegrated from the bone chip. In one sheep, the Fiber-Wire sutures were cut out of the bone chip and the tendon was again retracted. There were no revisions or reoperations prior to their failure in these four sheep.

## Discussion

As a result of previous studies [7-9,12], it appears that without restoration of the normal architectural structure of the muscle, physiological mechanical muscle function can not be restored. Restoration of muscle architecture is not possible in a single stage procedure, but might be possible with slow continuous traction [18,19].

Because progressive musculotendinous lengthening of a chronically retracted, musculotendinous unit has not previously been performed, a new method was needed. The present concept was selected for various reasons: First, we preferred to perform lengthening by a given distance per day (one mm) rather than by pulling with a specific constant load, as this method has been successful in lengthening muscle in the Ilizarov technique of bone lengthening [17,26]. Muscle lengthening appears to be better controllable and technically easier to perform. Second, we preferred to attach the lengthening device to the scapula instead of the humerus. Thereby, passive or active movement of the leg were not compromised, and movements of the animals’ shoulders did not influence the amount of lengthening of the musculotendinous unit. Furthermore, this method avoided the need to suspend the animals with belts during the time of muscle lengthening. To transmit the force to the body of the tensioner through a rotating, transcutaneous axis instead of a subcutaneous push/pull or rotating device allowed satisfactory control of the elongation process and the force which needed to be applied to exert further traction.

The mechanical in-vitro analyses documented, that the apparatus is capable of delivering adequate tension to the musculotendinous unit during the extrusion of the rod and should be capable of resisting loads, which were exerted by in-vivo contraction of the muscles. Previous in vivo force measurements of the sheep’s infraspinatus muscle have shown that the contractile strength affected by fatty infiltration was constantly between 50 and 200 N, with a maximum force of 310 N [7,24,27]. Forces counteracting the extrusion of the tread rod are the reflectory or active muscle contraction, the passive resistance of the stretched muscle fibers and surrounding scar tissue. The latter was removed during implantation of the tensioner as completely as possible. Intramuscular fibrous tissue however, was not removable and most likely the greatest source of resistance during the experiment.

Both tensioners showed in vitro enough fixation stability to counteract against the pullout strength of the muscle force. However, subsequent to cyclic loading the maximal resistance force was significantly lower in tensioner No. 1, which could be related to the increased fixation points of tensioner No. 2 with 6 screws. This may be a reason of failure by cut out of the tensioners’ body out of the scapular spine in four sheep.

Nevertheless, in vivo lengthening of a musculotendinous unit up to its’ original length was feasible in two thirds of the sheep. Despite the fact, that reversion of the original length of a chronic retracted rotator cuff tear could be achieved, the in-vivo experiments, however, proved to be difficult and several problems occurred which were specific to the in-vivo situation: Although the four sheep with cut outs were revised using tensioner No. 2, the intra experiment tension of the musculotendinous unit remained at the same level before and after revision. None of the tensioners No. 2 showed any complication with fixation on the scapular bone or breakage of the Nitinol® rotating axis. To avoid material failure in future, the tensioners’ body should be fixed using at least six fixed angle screws and a diameter of at least 2 mm of the Nitinol® rotating axis is needed.

Furthermore, infection occurred along the transcutaneous rotating axis extending to the body of the tensioner in three cases. In terms of animal protection in this demanding animal model, it is of tremendous importance to avoid complications with infections in future studies. Although we were able to standardize this model we think, a significant improvement of the method may be achieved if the force transmission to the tensioner would be performed through a short, subcutaneous mechanism, which is handled through the intact sheep’s skin. As study setup, surgeries and especially postoperative care were shown to be very demanding with a considerable rate of intraoperative and postoperative challenges, it is important that this device and animal model is used only by experienced surgeons and groups with intimate knowledge in handling sheep with postoperative care.

## Conclusion

This is the first series of experiments in which a mechanical device has been introduced for elongation and restoration of the original length of the musculotendinous unit. In future experiments, the functional and physiological properties of the muscle will be assessed during the phase of retraction and after lengthening, to evaluate if reversion of the muscle architecture does indeed result in an according restoration of the muscle’s physiological contractile properties, the final goal that this ongoing project and tendon repair in general are pursuing.

## Authors contributions

MAZ and EF and DCM carried out the experiments, participated in the design of the study, performed the statistical analysis and drafted the manuscript. BvR carried out the surgical interventions. RF designed and manufactured the tensioner, CG and DCM conceived of the study, and participated in its design and coordination and helped to draft the manuscript. All authors read and approved the final manuscript.
